# Involvement of Matricellular Proteins in Cellular Senescence: Potential Therapeutic Targets for Age-Related Diseases

**DOI:** 10.3390/ijms25126591

**Published:** 2024-06-15

**Authors:** Motomichi Fujita, Manabu Sasada, Takuya Iyoda, Fumio Fukai

**Affiliations:** 1 Department of Molecular Patho-Physiology, Faculty of Pharmaceutical Sciences, Tokyo University of Science, 2641 Yamazaki, Noda 278-8510, Chiba, Japan; 2 Clinical Research Center in Hiroshima, Hiroshima University Hospital, 1-2-3 Kasumi, Minami-Ku, Hiroshima 734-8551, Japan; 3 Department of Pharmacy, Faculty of Pharmaceutical Sciences, Sanyo-Onoda City University, 1-1-1 Daigaku-Doori, Sanyo-Onoda 756-0884, Yamaguchi, Japan

**Keywords:** matricryptins, matricryptic site, senescence-associated secretory phenotype, senotherapy, senomorphic drug, communication network factor, tenascin-C, PAI-1, osteopontin, periostin

## Abstract

Senescence is a physiological and pathological cellular program triggered by various types of cellular stress. Senescent cells exhibit multiple characteristic changes. Among them, the characteristic flattened and enlarged morphology exhibited in senescent cells is observed regardless of the stimuli causing the senescence. Several studies have provided important insights into pro-adhesive properties of cellular senescence, suggesting that cell adhesion to the extracellular matrix (ECM), which is involved in characteristic morphological changes, may play pivotal roles in cellular senescence. Matricellular proteins, a group of structurally unrelated ECM molecules that are secreted into the extracellular environment, have the unique ability to control cell adhesion to the ECM by binding to cell adhesion receptors, including integrins. Recent reports have certified that matricellular proteins are closely involved in cellular senescence. Through this biological function, matricellular proteins are thought to play important roles in the pathogenesis of age-related diseases, including fibrosis, osteoarthritis, intervertebral disc degeneration, atherosclerosis, and cancer. This review outlines recent studies on the role of matricellular proteins in inducing cellular senescence. We highlight the role of integrin-mediated signaling in inducing cellular senescence and provide new therapeutic options for age-related diseases targeting matricellular proteins and integrins.

## 1. Introduction

Cellular senescence is a physiological and pathological cellular process triggered by various cellular stresses, such as telomere shortening, aberrant oncogene activation, and DNA damage accumulation [1]. Senescent cells are characterized by stable proliferation arrest but remain viable and metabolically active. These cells are found not only in aged tissues, but also in embryonic development, wound healing, tissue remodeling, inflammatory lesions, and the tumor microenvironment [1]. Senescent cells can secrete a number of factors, such as chemokines, growth factors, proinflammatory cytokines, proteases, and lipids, collectively known as the senescence-associated secretory phenotype (SASP) [2,3]. The biological functions of the SASP exhibit beneficial and detrimental consequences in a context-dependent manner; transient SASP activity shows beneficial roles in human health, whereas dysregulated and long-lasting SASP activity plays crucial roles in the deleterious effects of age-related diseases [4]. Accordingly, mechanistic analyses of the cellular senescence and SASP development observed in the microenvironments of various diseases have been vigorously conducted in recent years [2]. Recent evidence shows that the onset and progression of age-related diseases, such as intervertebral disc degeneration (IDD) [5,6,7], osteoarthritis (OA) [8,9,10,11], and abdominal aortic aneurysm [12,13,14], are correlated with large numbers of senescent cells within the lesion. 

Cellular senescence is induced by the repeated exposure of cells to a variety of stimuli, such as chemotherapeutic drugs, oncogene activation, the exhaustion of cellular replicative potential, and oxidative stress [2]. Regardless of the cause, senescent cells are commonly observed to exhibit a characteristic flattened and enlarged morphology. However, it remains unclear whether this change in cell morphology during the process of cellular senescence is necessary for cellular senescence or merely a consequence. Several studies have provided important insights into pro-adhesive properties of cellular senescence [15,16,17], suggesting that cell adhesion to the extracellular matrix (ECM), which is involved in characteristic morphological changes, may play pivotal roles in cellular senescence. Cell adhesion to the extracellular matrix (ECM) is one of the important determinants of cell morphology, and adhesion receptor integrins are primarily responsible for this binding [18]. Unlike ECM molecules that act as structural components, secreted non-structural ECM components, called matricellular proteins, serve to modulate cell–ECM interactions by interacting with cell adhesion receptors, including integrins [19]. Families of matricellular proteins include centralized or cellular communication network (CCN), periostin, thrombospondin-1 (TSP-1), follistatin-like 1 (FSTL1), osteopontin (OPN), galectin-3 (Gal-3), plasminogen activator inhibitor-1 (PAI-1), pigment epithelium-derived factor (PEDF), and tenascin-C (TNC) [20]. Matricellular proteins exhibit versatile functions, which are governed by alternative splicing to generate multiple isoforms [21] or by proteolytic processing to liberate their bioactive fragments, termed matricryptins [22]. Moreover, matricellular proteins are typically expressed at high levels in developing tissues and show reduced expression in adult tissues [19]. Their expression levels are dysregulated in pathological states, including inflammation and tumorigenesis and in response to different types of injury, inflammation, or tumor development [19]. Therefore, matricellular proteins are considered to play major roles in the pathogenesis of age-related diseases. However, the substantial role of the matricellular proteins responsible for the onset and progression of age-related diseases has not been clarified. Recently, matricellular proteins have been shown to be able to induce cellular senescence [23,24,25], further supporting the belief that matricellular proteins are involved in the onset of age-related diseases. This also raises expectations that matricellular proteins may be a promising target for the treatment of such diseases.

In this review, we describe the recent studies on the role of matricellular proteins in inducing cellular senescence (Table 1). We also highlight the role played by integrin-mediated signaling in inducing cellular senescence and provide new therapeutic options for age-related diseases targeting matricellular proteins and their cell surface receptor integrins. Among matricellular proteins, we pay special attention to TNC and its peptide fragments. As detailed below, a peptide called TNIIIA2, derived from TNC, has been shown to cause cellular transformation and its malignant progression through various effects, including the induction of cellular senescence. 

## 2. Matricellular Proteins in Cellular Senescence

### 2.1. Centralized or Cellular Communication Network (CCN)

The CCN family comprises six homologous members (CCN1-6) [51]. CCN proteins contain four modules, namely insulin-like growth factor-binding protein (IGFBP) module, von Willebrand factor type C (vWC) module, TSP-1 module, and C-terminal cysteine knot (CT) (with CCN5 lacking the CT module) [52,53,54,55]. The distinct functional modules possessed by each member dictate their interaction with various binding ligands, such as integrins, leading to diverse biological roles of CCN proteins as matricellular proteins.

CCN1, also known as cysteine-rich protein 61 (CYR61) and IGFBP-10, is expressed in multiple tissues [52]. Its expression changes in response to endogenous factors and external stimuli, such as oxidative stress, mechanical stress, and ultraviolet irradiation [56,57,58,59], and aberrant CCN1 expression is associated with aging and age-related diseases, including idiopathic pulmonary fibrosis (IPF) [60] and OA [27,61,62]. Jun and Lau found that human fibroblasts stimulated in the presence of recombinant CCN1 exhibit irreversible growth arrest, an enlarged and flattened cell morphology, the expression of senescence-associated (SA)-β-gal (a lysosomal enzyme used widely as a marker of senescence), p53 and p16 (a commonly used marker of senescence-associated growth arrest), and the production of SASP factors, indicating the induction of cellular senescence [23]. The fibroblasts that adhered to plates coated with CCN1 also exhibited the characteristics of cellular senescence [23]. The authors suggested, using *Ccn1^dm/dm^* knockin mice carrying the integrin α6β1 binding-defective mutant, that cellular senescence induced by CCN1–integrin α6β1 interactions might restrict fibrosis during tissue repair [23]. Feng and colleagues found that age is positively correlated with the disease severity of OA and the expression levels of senescence marker p16 and CCN1 in human chondrocytes [27]. CCN1 is also able to induce cellular senescence in human chondrocytes [27], and the suppression of CCN1-induced chondrocyte senescence reduces OA severity in an age-related OA mouse model [27], suggesting that inhibition of cellular senescence caused by CCN1 might be a novel therapeutic strategy for treating OA.

CCN2 is additionally called connective tissue growth factor (CTGF) and IGFBP-8 [63]. CCN2 is expressed in a variety of tissues at the development stages, with particularly high levels in cartilage and vasculature [64], and its expression is maintained in normal adult tissues [59,65]. Furthermore, its expression levels are dysregulated in aging [66,67] and age-related diseases, including IPF [68], and liver fibrosis [69,70]. Thus, CCN2 could play substantial roles in the development and progression of fibrotic diseases [71]. Jun and Lau have found that, similar to CCN1 [23], CCN2 also induces fibroblast senescence by binding to integrin α6β1, revealing that CCN2-induced fibroblast senescence concurrently restrains fibrotic responses [24].

CCN3, also known as nephroblastoma overexpressed (NOV) and IGFBP-9 [63,72], is highly expressed in adult endotheliocytes, smooth muscle cells, fibroblasts, and chondrocytes [73]. Several studies have detected dysregulated expression of CCN3 in aging [32] and a variety of age-related diseases, including OA [74]. The elevated expression of CCN3 in articular cartilage is correlated with OA severity [74]. In OA samples with elevated levels of CCN3, the expression levels of p16 and IL-1β, a proinflammatory SASP, are increased [74]. Kuwahara and colleagues found that the exogenous addition of CCN3 induces increased expression levels of senescence-associated factors p21 and p53 in mouse articular chondrocytes [32]. Moreover, cartilage-specific CCN3-overexpressing mice show degradative changes in knee joints, accompanied by increased expression levels of senescence-associated factor p21 and IL-6, IL-8, and TNF-α, which are not only commonly used SASPs, but also proinflammatory degenerative genes [32]. Collectively, CCN3-induced cellular senescence might be partially involved in the pathology of OA. Further studies are expected regarding the substantial role of CCN3 in the pathology of OA. In addition, trophoblast cells, upon acquisition of senescent traits induced by CCN3, exhibit activation of focal adhesion kinase (FAK) and elevated cell migration, indicating that CCN3-enhanced adhesive activity is involved in the induction of cellular senescence [31]. 

CCN4, known alternatively as WNT1 inducible signaling pathway protein 1 (WISP1) [55], exhibits elevated expression in the cartilage of OA patients compared with controls, with expression strongly correlated with disease severity [33,75]. Unlike other CCNs, the exogenous addition of recombinant CCN4 suppresses the induction of cellular senescence and chondrocyte apoptosis in human OA [33]. The suppression of cellular senescence and chondrocyte apoptosis by CCN4 is blocked by treatment with anti-integrin αvβ3 antibody [33]. Interestingly, anti-integrin αvβ3 antibody alone is able to induce the cellular senescence of chondrocytes [33]. Furthermore, in the in vivo setting, injection of CCN4 small interfering (si)RNA into the knee joints of a rat model of OA increases disease severity, accompanied by an increased number of SA-β-gal-positive cells and apoptotic cells, compared with non-targeting siRNA [33], suggesting that CCN4 suppression of cellular senescence might be beneficial for OA. However, it should be noted that some reports suggest that CCN4 may demonstrate detrimental effects on the progression of OA [76,77]. 

### 2.2. Periostin

Periostin belongs to the fasciclin family [78]. Under physiological conditions, periostin is expressed in limited numbers of tissues, such as periodontal ligament and periosteum [79]. On the other hand, periostin is commonly overexpressed in human tissues during pathological processes, including IDD [80,81,82]. The expression levels of periostin in human IDD tissues are correlated with the severity of the condition [34,80]. Intervertebral discs comprise the highly hydrated and gelatinous nucleus pulposus (NP) core, the peripherally located multilaminar annulus fibrosus and the cartilaginous endplates, and their constituent cells may play a key role in the pathogenesis of IDD [83]. In the context in which abnormal mechano-stress is considered a risk factor for IDD [84], Wu and colleagues found that mechano-stress induces NP cell senescence [34]. Unbiased RNA sequencing and an assay of transposase-accessible chromatin sequencing revealed that mechano-stress induced NP cell senescence and the SASP upregulated periostin transcription [34]. Furthermore, periostin directly accelerated NP cell senescence by binding to integrin αVβ3, forming the self-amplifying loop of cellular senescence [34]. Periostin-induced cellular senescence is also observed in human annulus fibrosus cells [34]. In an in vivo analysis using a rat IDD model, the degenerative phenotype was attenuated by inhibition of periostin [34]. Similar observations were reported by Zhu and colleagues [81]. Collectively, periostin targeting might be beneficial for IDD treatment.

### 2.3. Thrombospondin-1 (TSP-1)

While the expression levels of TSP-1 are normally low in the healthy state, its expression levels are positively correlated with age [36,85]. Furthermore, its dysregulated expression has been associated with several age-related diseases, such as heart failure [86], glioblastoma [87], age-related macular degeneration [88], and OA [89]. An excellent review has already described the relationship of TSP-1 with aging and age-related diseases (reviewed in [90]). Please refer to the review for information on the alterations in TSP-1 expression and its biochemical functions in aging and age-related diseases. 

With regard to the induction of cellular senescence, several research papers suggest that TSP-1 directly induces cellular senescence in endothelial cells (ECs). Bitar reported that wound ECs derived from a diabetic rat model exhibit a characteristic feature of cellular senescence [91]. The cellular senescence induced by TSP-1 is thought to rely on its binding to its receptor CD47 [91]. Supporting this observation, Meijles and colleagues reported that TSP-1-induced cellular senescence in human pulmonary artery ECs occurs at a TSP-1 concentration within the range detected in the plasma of diseased patients [36,92,93]. Moreover, Gao and colleagues found that mouse ECs lacking CD47 have delayed senescence, enhanced proliferation, and increased tube formation in vitro and enhanced angiogenesis in vivo [35]. These results suggest that the TSP-1–CD47 signaling pathway positively regulates cellular senescence in ECs and may be a promising target for senotherapy in vascular diseases [94]. 

In addition, Baek and colleagues reported that TSP-1 deficiency leads to decreased survival and increased cancer aggression in a mouse model of Kras-induced lung tumorigenesis [37]. Mechanistically, in this mouse model, TSP-1 deficiency attenuated the senescence of lung lesions in lung adenomas [37]. Moreover, TSP-1 reduced the activation of pro-proliferative properties and maintained oncogenic Ras activation-induced senescence by tethering activated phospho-ERK to the cytosol [37], indicating that TSP-1 is involved in oncogenic-induced senescence.

### 2.4. Follistatin-like 1 (FSTL1)

FSTL1, additionally called follistatin-related protein and transforming growth factor (TGF)-β-stimulated clone 36 (Tsc36), was initially identified as a TGF-β-inducible gene [95], and is a secreted glycoprotein that belongs to the FS-SPARC family [96,97]. FSTL1 is considered to be strongly involved in age-related diseases (reviewed in [98]). Lung tissue and blood samples of IPF exhibit increased expression of FSTL1 compared with healthy controls [38,99,100]. Furthermore, neutralizing antibodies to functionally block FSTL1 and deletion of the FSTL1 gene attenuate bleomycin-induced lung fibrosis in mice [99,100]. However, the mechanisms underlying the detrimental effects of FSTL1 on the progression of pulmonary fibrosis have largely remained elusive. 

A growing body of evidence supports the idea that cellular senescence, especially in alveolar type II (ATII) cells, plays key roles in IPF development or progression [101,102,103]. Sun and colleagues revealed that FSTL1 alone promotes cellular senescence in human ATII cells, and FSTL1 administered via intratracheal routes induces lung fibrosis in mice, accompanied by increased cellular senescence [38], indicating that FSTL1-induced ATII senescence might contribute to the pathogenesis of IPF. 

In addition, FSTL1 increases with age in the NP of mice, and heterozygous FSTL1 knockout mice exhibit a reduced degree of degeneration compared with wild-type mice in a mouse model of IDD [104]. More recently, Yan and colleagues reported that the expression levels of FSTL1 are positively correlated with the disease severity of human IDD [39]. Mechanistically, recombinant human FSTL1 directly induces cellular senescence, accompanied by upregulated expression of proinflammatory SASP factors in NP cells derived from human IDD tissue [39]. In addition, FSTL1 silencing by siRNA ameliorates IDD progression and decreases the numbers of p16- and IL-1β-positive cells in the rabbit IDD model [39]. Taken together, targeting of FSTL1-induced senescence might be a novel therapeutic strategy for IDD.

### 2.5. Osteopontin (OPN)

OPN, also known as secreted phosphoprotein 1, bone sialoprotein I, and early T-lymphocyte activation-1, belongs to the small integrin-binding ligand N-linked glycoprotein family [105]. OPN levels increase with aging [106,107,108,109], and high expression levels of OPN are observed in age-related diseases, including OA [110]. The increase in OA disease severity is positively correlated with elevated expression levels of OPN, its receptor integrin αvβ3, and SA-β-gal [111]. In line with these observations, analysis of single-cell RNA sequencing has revealed that OPN is highly expressed in OA cartilage, and these cells possessed more senescent cell characteristics [110]. In contrast, Tian and colleagues have reported decreased expression levels of OPN in human primary chondrocytes of OA cartilage in comparison with normal human primary chondrocytes [40]. Furthermore, the depletion of OPN by siRNA enhances cellular senescence and proinflammatory mediators in human OA chondrocytes [40]. In an in vivo rat model of OA, the depletion of OPN by siRNA results in worse disease severity, accompanied by an increase in senescent cells, compared with non-targeting siRNA [40]. These results suggest that OPN plays a crucial role in modulating human chondrocytes from the inflammatory environment. Due to conflicting research results, further work is required to determine the biological function of OPN and disease development/progression of OA.

### 2.6. Galectin-3 (Gal-3)

Gal-3 belongs to the galectin family of lectins that binds galactose-containing glycoproteins [112]. Gal-3 is normally localized to the cytoplasm, but it can be secreted to the extracellular space and function as a matricellular protein [113,114]. The elevated expression of Gal-3 has been reported in various age-related diseases [115], including non-alcoholic steatohepatitis [116], OA [117], neurodegenerative diseases [118], heart failure [119], fibrotic diseases [112], and several types of cancer [120], and the expression levels of Gal-3 correlate with disease severity, such as IDD [121], suggesting its role in the onset or progression of age-related diseases.

Vlachou and colleagues have revealed that, although a mouse model of cardiomyopathy exhibits cardiac fibrosis, the Gal-3 deficiency in this model confines the profibrotic responses [122]. Mechanistically, the cardiac fibroblasts with deficient Gal-3 in this model show elevated proliferative potential and a decreased number of SA-β-gal-positive cells [122], indicating that Gal-3 might be involved in cardiac fibrosis and the promoting effect of Gal-3 on cellular senescence. 

In contrast, Gal-3 depletion has been associated with decreased proliferative potential and increased numbers of SA-β-gal-positive mouse fibroblasts, human foreskin fibroblasts, and human gastric cancer cells [41]. In in vivo settings using mice bearing human gastric cancer xenografts, the gastric tumor size was reduced in Gal-3-depleted xenografted mice [41], suggesting that Gal-3 suppresses premature senescence and aggravates gastric tumorigenesis. Collectively, Gal-3-induced cellular senescence might be quite diverse and cell type- and context-dependent.

### 2.7. Plasminogen Activator Inhibitor-1 (PAI-1)

PAI-1 is alternatively called serpin peptidase inhibitor, clade E member 1 (SERPINE1). In fibrinolysis, plasminogen activators (PAs) convert inactive plasminogen into plasmin, which ultimately enables the breakdown of fibrin clots. Conversely, PAI-1 inhibits PAs to reduce the generation of plasmin [123]. In addition, while plasmin has the ability to activate matrix metalloproteinase (MMP) and both plasmin and MMP can degrade ECM, the reduction in plasmin through PA inhibition by PAI-1 induces ECM deposition [123]. Besides its fibrinolytic function, matrix-bound PAI-1 can modulate the activity of cell adhesion via PA receptor [124,125].

An excellent review has already described the relationship between PAI-1 and senescence (reviewed in [126]). Notably, PAI-1 deficiency delays the induction of senescence and protects against organ dysfunction, leading to a prolonged lifespan of Klotho-deficient mice that exhibit a premature aging syndrome [127]. Furthermore, Kortlever and colleagues revealed that MEFs derived from PAI-1 knockout mice proliferate beyond the checkpoint of replicative senescence [43]. The authors suggest that PAI-1 plays a role in inducing replicative senescence as a downstream target of p53 [43]. Moreover, Elzi and colleagues revealed that PAI-1 and IGFBP3 each have the ability to induce cellular senescence [44]. IGFBP3-induced cellular senescence is suppressed by treatment with tissue-type PA (tPA) through the proteolysis of IGFBP3, and PAI-1 stabilizes IGFBP3 by inhibiting tPA-mediated proteolysis of IGFBP3, leading to induction of cellular senescence [44]. In addition, Omer and colleagues reported that the formation of stress granules in the proliferative state impairs the induction of fibroblast senescence by interfering with the pro-senescence function of PAI-1 [45]. Mechanistically, the translocation of PAI-1 to stress granules could inhibit PAI-1-induced cellular senescence by interfering with its secretion [45]. 

PAI-1-induced cellular senescence may contribute to the progression of age-related diseases, with stimulation with PAI-1 alone inducing cellular senescence in ATII cells [46]. Furthermore, bleomycin-induced ATII cell senescence is associated with an increase in PAI-1 expression and is suppressed by the genetic or pharmacological inhibition of PAI-1 [46]. In vivo studies have revealed that depletion of PAI-1 in ATII cells in mice attenuates bleomycin-induced lung fibrosis [46]. Thus, PAI-1-induced cellular senescence might play a role in the development of lung fibrosis. 

Moreover, PAI-1 is highly expressed in human atherosclerotic plaques and positively correlated with the expression of the senescence marker p21 [47]. In the context where senescence of coronary artery smooth muscle cells (SMCs) plays a significant role in atherogenesis [128,129], PAI-1 induces the cellular senescence of human coronary artery SMCs by binding to LRP1, which is a PAI-1 receptor. PAI-1-induced cellular senescence is inhibited by a PAI-1 inhibitor and by an antibody against LRP1 [48]. In in vivo settings, PAI-1 inhibitors attenuate atherosclerosis formation in a mouse model of atherosclerosis [48]. Taken together, the PAI-1–LRP1 axis at least partly promotes the development of atherosclerosis through the induction of cellular senescence. 

### 2.8. Pigment Epithelium-Derived Factor (PEDF)

PEDF, additionally called early population doubling cDNA-1 and SERPINF1, belongs to the SERPIN family. PEDF is a secreted glycoprotein that exhibits anti-angiogenic, anti-oxidant, anti-tumorigenic, and neuroprotective properties [130,131,132,133]. PEDF is associated with aging and age-related diseases (reviewed in [132]), and, additionally, has effects on mesenchymal stem cells (MSCs). MSCs have considerable plasticity and represent a promising candidate for various clinical applications in the field of regenerative medicine [134]. To successfully apply MSCs to clinical applications, MSCs must be expanded. However, the expansion of MSCs is limited by irreversible growth arrest and cellular senescence. PEDF is able to reduce oxidative stress [135] and, based on its ability, PEDF delays cellular senescence in MSCs and allows for greater cell expansion [49], allowing their use as promising tools for regenerative therapy. On the other hand, PEDF expression has been reported to be increased in MSCs derived from old mice, and the expression of PEDF is associated with decreased efficacy of MSCs in treating myocardial infarction in a mouse model [136]. 

### 2.9. Tenascin-C (TNC)

TNC is abundantly expressed during embryonic development and around birth before decreasing in adulthood, when expression is at relatively low levels and restricted to sites with high cell turnover [137]. The versatility of TNC function is thought to be based not only on differences in receptors and the presence of multiple alternative splicing variants, but also its context-dependent liberation via proteolytic cleavage by MMP and a disintegrin and metalloproteinase with a TSP motif [138,139]. Aberrant TNC expression is associated with aging [140,141,142] and the pathogenesis of several age-related diseases, including OA [143], heart disease [144], fibrosis [145], and cancer [146]. However, the substantial role of TNC in age-related diseases remains largely unclear.

Proteolytic degradation of TNC has been detected in lung and colon cancer, and early-stage non-small-cell lung cancer patients with TNC degradation show significantly worse prognosis and higher recurrence than those without TNC degradation [147,148,149]. Increased MMP activity has been observed in patients with degraded TNC, indicating that exposure of the TNC functional cryptic site by several inflammatory proteinases may be associated with the malignant progression of cancer [147]. Among the TNC variants, those containing the FNIII A2 repeat are highly expressed in malignancies [150]. The variants containing FNIII A1A2 repeats also inhibit T cell activation in vitro, suggesting that TNC exerts immunosuppressive activity [151]. Additionally, work from our laboratory has shown that TNC harbors a functional cryptic site that comprises the YTITIRGV amino acid sequence located in the FN type III A2 repeat, and fragments containing this sequence, termed TNIIIA2 peptide, can potently enhance cell adhesion by activating β1-integrins; this activation state is sustained for an extended period [152]. Based on these functions, TNIIIA2 contributes to the ability of cancer cells to acquire malignant properties, including hyper-proliferation, disseminative migration, anchorage-independent growth, and anoikis resistance [153,154,155,156,157]. More recently, we found that TNIIIA2 peptide induces cellular senescence in human fibroblasts through β1-integrin activation [25]. In the induction of cellular senescence by TNIIIA2, it was particularly important to maintain the strongly activated state of integrin by TNIIIA2 for several days. This abnormal state of cell adhesion by TNIIIA2 is thought to cause ROS production, resulting in cellular senescence. Moreover, the humoral factors secreted by TNIIIA2-induced senescent fibroblasts cause preneoplastic epithelial cells to acquire malignant properties, perhaps leading to cancer progression [25]. Thus, our study demonstrates that the TNC molecule plays two functional roles that favor the malignant transformation of tumor cells. That is, TNIIIA2, as a TNC-derived matricryptin, not only directly acts on tumor cells to induce cancer aggressiveness, but also indirectly contributes to pro-tumorigenic effects through the induction of cellular senescence in fibroblasts. Functional blocking antibodies against TNIIIA2 [158] are expected to exhibit powerful anticancer effects against malignant tumors based on these two distinct functions. 

## 3. Potential Strategies Targeting Cellular Senescence by Blocking Matricellular Protein Function and Integrin Signaling Pathway 

### 3.1. Blocking Matricellular Protein Function as a Potential Senotherapeutic Strategy

Recently, clinical trials of agents targeting characteristics of cellular senescence, such as senolytic (killing senescent cells), senomorphic/senostatic (modulating phenotypes of senescent cells), or senostatic (modifying or suppressing the SASP) agents, are gradually accumulating insights into the relationship between senescent cells and human diseases [159]. Table 2 summarizes drugs targeting matricellular proteins that are under active clinical development and that are focused on in this review. Although no drugs have yet been approved that target cellular senescence by blocking matricellular proteins, clinical trials are underway for age-related diseases. 

In the preceding sections, we summarized the relationship between matricellular protein-induced cellular senescence and the pathogenesis of age-related diseases. While knockout mice of some matricellular proteins, including CCN1, CCN2, and FSTL1, are characterized by embryonic or neonatal lethality, knockout mice of most matricellular proteins exhibit no apparent post-natal phenotypic changes [19]. Considering that the expression of matricellular proteins is context-dependent, as mentioned above, it is conceivable that matricellular protein targeting is unlikely to increase pleiotropic adverse effects. Therefore, the targeting of matricellular proteins may be an attractive strategy. Further research to obtain a deep understanding of both the regulatory mechanisms of matricellular proteins regarding the induction of cellular senescence and their associated pathogenesis in age-related diseases is required for clinical applications.

### 3.2. Role of Integrin-Mediated Cell Adhesion in Cellular Senescence 

As mentioned in the above sections, cellular senescence directly induced by several matricellular proteins is clearly mediated by integrins. Senescent cells generally display a flattened and enlarged cell morphology, regardless of the stimuli inducing the senescence [178]. This suggests that integrin-mediated signaling may play a role in the induction of cellular senescence. Indeed, multiple studies have reported that integrin-mediated signaling is directly associated with cellular senescence induction (Table 3).

Shin and colleagues reported that the downregulation of p21-activated kinase-interacting exchange factor-β (βPIX), a negative regulator of focal adhesion [191], induces fibroblast senescence through enhanced cell adhesive activity; this is accompanied by increases in focal adhesions and actin stress fibers. This cell adhesion-induced cellular senescence is abolished by treatment with arginyl-glycyl-aspartic acid (RGD) peptide or FAK inhibitor [17].

ROS generated within cells are the main cause of stress-induced cellular senescence [192]. Intracellular ROS is produced by FAK–Rac1 activation following integrin engagement through fibronectin–α5-integrin-mediated cell adhesion [193]. Furthermore, it has been reported that cells expressing the auto-clustering β1-integrin mutant have upregulated ROS production [194], indicating that the production of ROS resulting in the induced cellular senescence hinges upon the cell adhesive activity mediated by integrins. Indeed, the accumulation of ROS is required for the binding of CCN1 or CCN2 to β1-integrin in CCN1- or CCN2-induced senescent fibroblasts [23,24]. Our group also found that β1-integrin activation-induced cellular senescence mediated by TNC-derived peptide is caused by ROS production and the resulting DNA damage [25]. Interestingly, our experiments showed that cellular senescence is induced not by the mere transient activation of integrin, but by its continued activation for more than a few days [25]. Cell adhesion via activated integrins is an essential event for cell survival, but maintaining the activated state at a high level for a long period may involve considerable stress for cells. It is possible that other matricellular proteins capable of inducing cellular senescence may similarly cause strong and sustained activation of integrin, thereby inducing ROS production and consequent cellular senescence.

Changes in mechanical properties, the increase in ECM, and tissue stiffness contribute to disease development and progression [195,196,197,198]. Notably, many studies have demonstrated that tissue stiffening enhances integrin signaling [199,200], and increasing evidence on enhanced ECM stiffness-induced cellular senescence has provided important insights into the pathogenesis of age-related diseases, including OA, abdominal aortic aneurysm, and IDD [34,201,202,203,204,205]. Wang and colleagues recently found that the increased stiffness of human NP tissue specimens with IDD is correlated with an increase in the degeneration grade [203]. More recently, a stiff substrate has been reported to induce cellular senescence in NP cells, which is accompanied by upregulation of β1-integrins or activation of the mechanosensitive Piezo1 channel [34,202,203]. Matricellular proteins expressed in age-related diseases also increase ECM stiffness through integrin-mediated signaling pathways. Growing evidence regarding the correlation between ECM stiffness and malignant progression will offer valuable insights into the significant roles of TNC [206,207,208]. Barnes and colleagues have found that glioblastoma exhibits an increased TNC-enriched stiffened ECM and elevated integrin mechanosignaling. Furthermore, glioblastoma xenografts derived from cells expressing auto-clustering active mutants of β1-integrins exhibit enhanced integrin mechanosignaling, increased TNC-enriched stiffened ECM, and, ultimately, worse tumor aggressiveness [207]. Moreover, our group suggested that the TNC-derived matricryptin TNIIIA2 peptide might generate a tumor microenvironment exhibiting pro-adhesive activity via the aberrant activation of β1-integrins [25], possibly resulting in increased stiffness.

Although further studies are needed regarding the matricellular protein–integrin axis in the induction of cellular senescence, the targeting of integrins, which act as receptors for matricellular proteins, may also regulate cellular senescence, offering a promising strategy for the treatment of age-related diseases.

### 3.3. Integrin Modulators as a Potential Senotherapeutic Strategy

Recent studies have accumulated evidence that several integrin inhibitors have the ability to suppress the induction of cellular senescence and/or the acquisition of the SASP in cell-based assays and preclinical studies (Table 4). These inhibitors could be potential candidates for senotherapy in age-related diseases.

Most common integrin inhibitors are antagonists that competitively inhibit integrin–ligand binding. Unlike these antagonistic peptides, peptide FNIII14 inhibits integrin signaling by an entirely distinct mechanism; peptide FNIII14 induces a conformational shift in β1-integrins from the active to an inactive state, leading to functional inactivation [210,211]. Based on this unique activity, peptide FNIII14 exhibits therapeutic potential in animal models of several conditions, such as metabolic diseases, organ fibrosis, malignant tumors, and atherosclerosis [210,212]. Furthermore, in the previous section (see Section 2.9), we noted that TNIIIA2, a matricryptin of TNC, activates β1-integrins and induces cellular senescence in human fibroblasts [25]. In contrast, peptide FNIII14 inhibits the cellular senescence induced by TNIIIA2 [25]. Peptide FNIII14, due to its novel mechanism targeting integrins, might become a promising senotherapeutic agent. While one intrinsic drawback of peptide drugs is their low bioavailability due to their susceptibility to enzymatic degradation in vivo [213], technology for a drug delivery nanosystem for peptide FNIII14 suitable for in vivo applications has recently been developed [214]. Further progress in consideration of clinical applications is expected.

## 4. Conclusions and Perspectives

Senotherapeutics have shown promising results in alleviating age-related diseases in animal models [215] and, based on the results, several candidate senotherapeutics have been advanced into clinical trials [216]. However, senotherapeutic intervention strategies, including senolytic drugs, might have some concerning adverse effects, especially with long-term use [217]. Considering that the expression of matricellular proteins is context-dependent and that mice knockout models of most matricellular proteins exhibit no apparent post-natal phenotypic changes [19], the targeting of matricellular proteins for senotherapy is unlikely to result in deleterious pleiotropic adverse effects, making it an attractive strategy. Furthermore, integrin-mediated events, including cell adhesion and sensing of ECM stiffness, contribute to the induction of cellular senescence, suggesting the potential utility of integrin modulators targeting cellular senescence for the treatment of age-related diseases. The clinical development of integrin-targeted therapeutics has vigorously progressed and is marked by the recent approval of orally administered medications [218]. However, the relationship between integrin and the induction of cellular senescence is highly complex [219]; it appears that integrin-regulated cellular senescence is dependent on a multitude of factors, including cell type, stimulus, and integrin subunit. To develop integrin-targeted therapeutics for age-related diseases, more careful consideration of the consequences of integrin blocking, as well as rigorous preclinical applications, are essential.

## Figures and Tables

**Table 1:** Summary of major matricellular proteins in the induction of cellular senescence.

Matricellular Proteins	Binding Receptors	Roles in Cellular Senescence	Target Cell/Tissue	Possible Relevance to Disease Onset/Progression	Ref.
CCN1	Integrin α6β1	Induction	Wound granulation tissue of mouse skin, Human fibroblasts	Anti-fibrogenic response	[23]
	Integrin α6β1	Induction	Mouse model of CCl_4_ induced liver fibrosis, Hepatic stellate cells (Mouse, Human), Portal fibroblasts	Protective role in fibrosis	[26]
	ND	Induction	Human chondrocytes	Progression of OA	[27]
	Integrin α6β1	Induction	Human non-small-cell lung carcinoma cells	Decreased cancer aggressiveness	[28]
	ND	Induction	Mouse muscle progenitor cells	Progression of sarcopenia	[29]
	ND	Induction	Cerebral endothelial cells isolated from aged mice	-	[30]
	ND	Induction	Human cytotrophoblast cell	Modulation of preeclampsia	[31]
CCN2	Integrin α6β1	Induction	Wound granulation tissue of mouse skin, Human fibroblasts	Anti-fibrogenic response	[24]
CCN3	ND	Induction	Mouse articular chondrocytes	Modulation of OA	[32]
	ND	Induction	Human cytotrophoblast cell	Modulation of preeclampsia	[31]
CCN4	Integrin αVβ3	Suppression	Human chondrocytes isolated from knee OA	Protective role in OA	[33]
Periostin	Integrin αVβ3	Induction	Human nucleus pulposus cells, Human annulus fibrosus cells	Progression of IDD	[34]
TSP-1	CD47	Induction	Mouse endothelial cells	Modulation of endothelial dysregulation	[35]
	CD47	Induction	Human pulmonary artery endothelial cells, Human aortic endothelial cells, mouse lung	-	[36]
	ND	Induction	Mouse fibroblasts, Epithelial cells from LSL-KRAS^G12D^ mice, Mouse model of Kras^G12D^-mediated lung tumorigenesis	Decreased cancer aggressiveness	[37]
FSTL1	ND	Induction	Human alveolar type II cells	Progression of pulmonary fibrosis	[38]
	TLR4	Induction	Human nucleus pulposus cells tissues from IDD, Rabbit IDD model	Progression of IDD	[39]
OPN	ND	Suppression	Human articular chondrocytes, Rat knee joint cartilage	Protective role in OA	[40]
Gal-3	ND	Suppression	Mouse embryonic fibroblasts, Human foreskin fibroblasts, Human gastric carcinoma cell lines	Decreased cancer aggressiveness	[41,42]
PAI-1	ND	Induction	Human fibroblasts, Mouse fibroblasts, Human breast cancer cells, Human retinal pigment epithelial cells	-	[43,44,45]
	ND	Induction	Alveolar type II cells (Rat, Mouse)	Progression of pulmonary fibrosis	[46]
	ND	Induction	Human umbilical vein endothelial cells	Progression of atherosclerosis	[47]
	LRP1	Induction	Human coronary artery smooth muscle cells, Mouse aorta	Progression of atherosclerosis	[48]
PEDF	ND	Suppression	Human mesenchymal stem cells	Expansion of MSCs	[49]
	ND	Suppression	Mouse retinal pigment epithelium	-	[50]
TNC	β1-integrin	Induction	Human fibroblasts	Increased cancer aggressiveness	[25]

CCN, centralized or cellular communication network; TSP, thrombospondin; FSTL1, Follistatin-like 1; OPN, osteopontin; Gal, galectin; PAI, plasminogen activator inhibitor; PEDF, pigment epithelium-derived factor; TNC, tenascin-C; TLR4, toll-like receptor 4; LRP, low-density lipoprotein receptor-related protein; CCl_4_, carbon tetrachloride; OA, osteoarthritis; IDD, intervertebral disc degeneration; MSC, mesenchymal stem cell; ND, not determined.

**Table 2:** Drugs targeting matricellular proteins under active clinical development and focused on in this review.

Agents	Mode of Action	Modality	Diseases	Phase	ID Number
Pamrevlumab (FG-3019)	Anti-CCN2	Antibody	IPF	Phase III	jRCT2051210169 [160]
			Pancreatic cancer	Phase III	NCT03941093 [161]
			Duchenne muscular dystrophy	Phase III	NCT04371666 [162]
SHR-1906	Anti-CCN2	Antibody	IPF	Phase II	NCT05722964 [163]
OLX-101A (BMT-101)	CCN2 expression inhibitor	siRNA	Hypertrophic scar	Phase II	NCT04877756 [164]
PRS-220	Anti-CCN2	Antibody Mimetics	IPF	Phase I	NCT05473533 [165]
LEM-S401	CCN2 expression inhibitor	siRNA	Hypertrophic scar	Phase I	NCT04707131 [166]
VT-1021	TSP-1 expression inducer	Peptides	Solid Tumors, Glioblastoma	Phase II/III	NCT03364400 [167]
belapectin	Gal-3 inhibitors	Polysaccharide polymer	NASH	Phase II/III	NCT04365868 [168]
proLectin-M	Gal-3 inhibitors	Carbohydrate polymers	COVID-19	Phase II	NCT04512027 [169]
TB-006	Anti-Gal-3	Antibody	Alzheimer’s disease	Phase II	NCT05476783 [170]
Selvigaltin (GB1211)	Gal-3 inhibitors	Small Molecules	Hepatic impairment	Phase I/II	NCT05009680 [171]
			Solid Tumors	Phase II	NCT05913388 [172]
TM5614	PAI-1 inhibitors	Small Molecules	CML	Phase III	jRCT2031220084 [173]
			Solid Tumors	Phase II	jRCT2061230039 [174]
			Cutaneous angiosarcoma	Phase II	jRCT2021230016 [175]
			SSc-ILD	Phase II	jRCT2021230022 [176]
DVC1-0401	PEDF expression inducer	Virus vector	Retinitis pigmentosa	Phase I/II	jRCT2073180024 [177]

CCN, cellular communication network; TSP, thrombospondin; Gal, galectin; PAI, plasminogen activator inhibitor; PEDF, pigment epithelium-derived factor; siRNA, small interfering RNA; IPF, idiopathic pulmonary fibrosis; NASH, nonalcoholic steatohepatitis; COVID-19, coronavirus disease 2019; CML, chronic myelogenous leukemia; SSc-ILD, systemic sclerosis-associated interstitial lung disease. Retrieved from ClinicalTrials.gov and Japan Registry of Clinical Trials, accessed on 10 March 2024.

**Table 3:** Roles of integrins in the induction of cellular senescence.

Integrins	Role in Cellular Senescence	Function	Ref.
β1-integrin	Induction	βPAK-interacting exchange factor induces cellular senescence in human diploid fibroblasts via persistent activation of β1-integrin	[17]
	Induction	Tenascin-C-derived peptides induce cellular senescence in human diploid fibroblasts via activation of β1-integrin	[25]
	Suppression	Specific deletion of β1-integrin in a mouse model of spontaneous insulinoma formation resulted in the acquisition of a senescence phenotype in a tumor nest of β tumor cells	[179]
	Suppression	Epithelial specific depletion of β1-integrin promotes cellular senescence in mammary intra-epithelial neoplasm lesions in a mouse model of luminal B human breast cancer	[180]
Integrin α2β1	Induction	Integrin α2β1 antagonistic peptide suppresses chemotherapy-induced senescence of human bladder cancer cells	[181]
	Suppression	Integrin α2β1 depletion causes cellular senescence in human melanoma cells	[182]
Integrin α5β1	Induction	Integrin α5β1 antagonist suppresses the ellipticine- or temozolomide-induced senescence of human glioblastoma cells	[183]
Integrin α6β1	Induction	CCN1 induces cellular senescence in human diploid fibroblasts by binding to integrin α6β1	[23]
	Induction	CCN1 induces cellular senescence in hepatic stellate cells and portal fibroblasts by binding to integrin α6β1	[26]
	Induction	CCN1 induces cellular senescence in non-small-cell lung carcinoma cells by binding to integrin α6β1	[28]
	Induction	CCN2 induces cellular senescence in human diploid fibroblasts by binding to integrin α6β1	[24]
β3-integrin	Induction	Increased expression of β3-integrins render mouse embryonic fibroblasts more susceptible to replicative senescence	[184]
	Induction	Increased expression of β3-integrins promotes replicative senescence and oncogene-induced senescence in human breast fibroblasts.	[185]
	Induction	β3-integrin overexpression induces cellular senescence in human tubular cells	[186]
Integrin αvβ3	Induction	Integrin αvβ3 antagonist inhibits periostin-induced senescence in human nucleus pulposus cells	[34]
	Suppression	Treatment with function-blocking integrin αvβ3 antibody induces senescence in human articular chondrocytes	[33]
	Suppression	β3-integrin depletion and treatment with the f function-blocking integrin αvβ3 antibody induce senescence in glioblastoma cells	[187]
β4-integrin	Induction	β4-integrin depletion inhibits senescence of human umbilical vascular endothelial cells	[188]
	Suppression	β4-integrin depletion induces senescence of human bronchial epithelial cells	[189]
Integrin α6β4	Induction	Integrin α6β4 dimerizes, is activated, and induces premature senescence in cancer cells when they are exposed to ionizing radiation	[190]

PAK, p21-activated kinase; CCN, cellular communication network.

**Table 4:** Integrin inhibitors capable of suppressing cellular senescence and/or SASP acquisition.

Agent	Mode of Action	Modality	Function	Refs.
RGD peptide	Integrin antagonist	Peptide	Suppression of βPAK-interacting exchange factor-induced cellular senescence in human fibroblasts	[17]
TFA peptide	Integrin α2β1 antagonist	Peptide	Suppression of chemotherapy-induced senescence of primary bladder cancer cells and the human bladder cancer cell line T24 in a 3D collagen I gel model and in T24-bearing mice	[181]
Cilengitide	Integrin αvβ3 and αvβ5 antagonist	Peptide	Reduction in the SASP secreted from oncogene-induced senescence in human fibroblasts	[185]
GSK3008348	Integrin αvβ6 antagonist	Small molecule	Reduction in the SASP secreted from bleomycin-induced senescence in organoids comprising human alveolar epithelial cells and human fibroblasts	[209]
T1 peptide	Integrin α6β1-binding peptide	Peptide	Suppression of CCN1-induced senescence in human fibroblasts	[23]
GoH3	α6-integrin neutralizing antibody	Antibody	Suppression of CCN1- or CCN2-induced senescence in human fibroblasts	[23,24]
SJ749	Integrin α5β1 antagonist	Small molecule	Suppression of ellipticine-induced cellular senescence in human glioblastoma cells	[183]
K34c	Integrin α5β1 antagonist	Small molecule	Suppression of ellipticine- or temozolomide-induced cellular senescence in human glioblastoma cells lines or human colon cancer cell lines	[183]
Peptide FNIII14	β1-integrin inactivator	Peptide	Suppression of TNC-derived peptide-induced cellular senescence in human fibroblasts	[25]
Isoliquiritigenin	β3-integrin inactivator	Small molecule	Inhibition of β3-integrin-mediated cellular senescence in HK-2 cells, and reduction in cellular senescence and interstitial fibrosis in the UUO mouse model	[186]

RGD, arginine-glycine-aspartic acid; PAK, p21-activated kinase; SASP, senescence-associated secretory phenotype; CCN, cellular communication network; TNC, tenascin-C; HK-2, human proximal tubule epithelial cell line; UUO, unilateral ureteral obstruction.

## Data Availability

Not applicable.
